# Analysis of the Rolling Process of Alloy 6082 on a Three-High Skew Rolling Mill

**DOI:** 10.3390/ma18112618

**Published:** 2025-06-03

**Authors:** Rail Sovetbayev, Yerik Nugman, Yerzhan Shayakhmetov, Yermek Abilmazhinov, Anna Kawalek, Kirill Ozhmegov

**Affiliations:** 1 Department of Mechanical Engineering, NJSC “Kazakh National Research Technical University Named after K.I. Satpayev”, Almaty 050000, Kazakhstan; e.nugman@satbayev.university; 2 Department of Digital Technologies in Mechanical Engineering and Logistics, Shakarim University, Semey 071412, Kazakhstan; shaiakhmeterzh@mail.ru (Y.S.); eras71@mail.ru (Y.A.); 3 Department of Production Management, Technical University Czestochowa, 42-201 Czestochowa, Poland; anna.kawalek@pcz.pl (A.K.); kvozhmegov@wp.pl (K.O.)

**Keywords:** aluminum alloy 6082, physical modeling, three-high skew rolling mill, rod rolling, numerical modeling, strength, plasticity

## Abstract

Modern requirements for aluminum alloys used in mechanical engineering and aviation include increased strength characteristics and refined microstructure. One of the promising methods for improving the properties of aluminum alloys is rolling on a three-high skew rolling mill, which provides intense plastic deformation and a fine-grained structure. This study describes the results of numerical modeling of the rolling process of aluminum alloy 6082 rods in a three-high skew-type mill. Numerical modeling of alloy 6082 was conducted using the ForgeNxT 2.1 software designed to simulate metal-forming processes, including rolling. The rheological behavior of the material under study was investigated by compression tests using a Gleeble 3800 plastometer (“DSI”, Austin, TX, USA), which enabled the determination of the main parameters of material flow under specified conditions. The process of rolling bars of alloy 6082 on a three-high skew mill was numerically analyzed in the temperature range of 350–400 °C. This allowed for the study of the distribution of stresses, temperatures, and strain rates from the rolling mode. A physical experiment was conducted to validate the results of numerical modeling. The obtained results enabled the identification of rolling modes that promote microstructure refinement and enhance the mechanical properties of the alloy.

## 1. Introduction

Aluminum alloys find widespread application in the fields of mechanical engineering, aviation, shipbuilding, and energy owing to their high strength, corrosion resistance, and favorable processability. In particular, alloy 6082 is in high demand for the production of load-bearing components in vehicles, power plant components, and building constructions [1,2,3]. However, conventional plastic processing techniques do not consistently yield a refined microstructure and superior mechanical properties, which may result in a reduced service life of these products.

Developing technological processes to obtain aluminum alloy 6082 products with predictable mechanical properties and a refined microstructure is an important task in modern metallurgy. This is particularly relevant given the growing requirements for materials used in critical structures, which demand a combination of high strength, plasticity, and corrosion resistance [4].

Fine-grained alloys meet these requirements and exhibit enhanced static and cyclic strength, hardness, and wear resistance. Such a microstructure can be achieved through severe plastic deformation (SPD) [5,6].

A key factor determining the application of severe plastic deformation in the manufacturing process is the ability to provide large deformations of the metal without failure. Several SPD methods exist, including equal channel angular pressing (ECAP) [7,8,9,10]. This method was developed by V. M. Segal in the early 1970s and further refined by R. Z. Valiev et al. in the early 1990s as a way to form ultrafine-grained structures [11,12].

The ECAP method involves deformation by forcing the workpiece through two intersecting channels at an angle ϕ, typically 90°, 120°, or 135°. This is a preferred approach for producing homogeneous ultrafine-grained structures with desirable mechanical properties across various materials [13,14]. The primary advantage of this method is the preservation of the original sample shape, enabling multiple pressing cycles. However, the ECAP method has limitations, such as restrictions on sample size (the sample length is typically limited to 150 mm or less) and difficulties in processing materials with low deformation capacity. For such materials with low deformation capacity, this problem was addressed by increasing the channel conjugation angle from 90° to 120° and elevating the pressing temperature [15].

An alternative method to SPD is the high-pressure torsion (HPT) technique. In HPT, the sample is compressed between Bridgman anvils under applied pressure (5–10 GPa). By rotating the movable anvil, a significant true logarithmic strain (ε > 5) is generated through elementary shear. Depending on the material and the strain value (ε), HPT can be used to obtain integral disk-shaped samples up to 20 mm in diameter with nanocrystalline or amorphous structures [15,16,17]. However, the small size of the produced samples limits the industrial relevance of this method, though it remains valuable for obtaining model materials to study nanocrystalline structures.

One of the SPD methods is accumulative roll bonding (ARB), a process that involves repeatedly gluing and rolling metal sheets to achieve an ultrafine-grained structure [18]. Despite their advantages, the industrial application of these severe plastic deformation methods (ECAP, HPT, and ARB) is limited by their high complexity of implementation, the restricted size of the resulting workpieces, and the inherent low productivity.

One effective technique for achieving a refined grain structure is the KOBO method, which involves the cold extrusion of metals through a die twisted on both sides. This process was developed at the AGH University of Science and Technology and can deform even materials produced via powder metallurgy [19]. Additionally, other industrial-scale approaches, such as all-round forging [20] and twist extrusion [21], have demonstrated the capability to fabricate ultrafine-grained materials under production conditions.

However, as we mentioned, these methods are limited by their complexity of implementation, the restricted size of the workpieces (which can limit their suitability for large-scale production), and other drawbacks. The KOBO method has high requirements for the quality of the source material, while all-round forging suffers from high energy costs and intense deformation that can lead to structural heterogeneity. In contrast, the method of deformation treatment on a three-high skew rolling mill (SRM) using significant roll inclination angles is promising and relevant from economic and technological perspectives. During three-high SRM rolling, a stress state close to all-round compression with pronounced shear deformation is formed in the metal. Optimizing the roll inclination angle can increase technological plasticity by over 300%. This method is employed in a wide range of processes, including the rolling of both steel and non-ferrous rods, and provides the necessary fine-grained structure.

Scientific novelty of the work:

- The conditions for intensive deformation treatment via the skew-type (radial-shear) rolling method to create a fine-grained structure in alloy 6082 rods have been determined;
- The rheological behavior of alloy 6082 has been studied for the first time to create a digital twin of the skew-type rolling process.

This study investigated the rolling of aluminum alloy 6082 on a three-high skew-type rolling mill, a process that belongs to the methods of severe plastic deformation. Optimizing and predicting the properties and structure of the final product through numerical modeling is crucial, as it helps to save time and resources for conducting expensive full-scale experiments. This study aims to identify the optimal parameters for rolling aluminum alloy 6082 rods on a three-high skew-type rolling mill, with the objective of refining the structure and enhancing the mechanical properties of the final product.

In order to accomplish the objective, the following research activities were performed:

- Rheological studies were conducted to investigate the flow behavior of the specified alloy, process the research results, and establish the relationship between deformation resistance and the parameters of thermomechanical deformation;
- A numerical model was developed and utilized to simulate the deformation processing of an aluminum alloy 6082 rod on a three-high skew rolling mill;
- The simulation results were experimentally verified in laboratory conditions with subsequent analysis of the structure and properties of the rolled material.

## 2. Materials and Methods

Aluminum alloy 6082 was selected as the subject material for this study. The chemical composition of this alloy is presented in Table 1, and its mechanical property characteristics are detailed in Table 2.

To conduct numerical modeling, in addition to the chemical composition, it is necessary to gather data on the material’s behavior during plastic deformation. This can only be achieved through physical modeling on test facilities under conditions as close to reality as possible. These tests are essential to determine the influence of temperature, speed, and degree of deformation on the deformation resistance (σ_p_), as well as the changes in the structure of the studied aluminum alloy 6082.

The metal’s resistance to plastic deformation, denoted as the flow stress (σ_p_), represents the stress required to initiate and sustain the plastic flow of the material. In the context of metal-forming processes, the current value of the flow stress (σ_p_) for a plastically deformed material is primarily influenced by the prevailing thermomechanical parameters (T, ε, and έ). The effects of these parameters on the flow stress (σ_p_) are interrelated and should be evaluated collectively [22].

Determining the flow stress, denoted as σ_p_, of the material under study is crucial when designing new plastic processing methods, employed in both hot and cold forming [23,24,25,26,27]. Accurately characterizing the material properties through stress–strain curves, accounting for the influences of temperature and strain rate, ensures enhanced precision in calculations using empirical formulas, as well as numerical simulations employing the finite element method.

In the available computer programs designed for modeling metal plastic processing using the finite element method, the flow stress values (σ_p_) are determined based on the adopted constitutive model. The metal’s resistance to plastic deformation, σ_p_, is most often described by a functional relationship σ_p_ = (ε, $\dot{\varepsilon }$, and T). Various mathematical functions have been proposed to model the changes in σ_p_ as a function of strain (ε), temperature (T), and strain rate ($\dot{\varepsilon }$) in the published literature [13,14,15,16].

The changes in the flow stress, σ_p_, are typically described using a function in the form of a constitutive Equation (1). This relationship is commonly employed to determine the value of σ_p_ in computer programs used for numerical modeling of plastic processing operations [28]:

(1) $$\sigma_{p}=A\cdot e^{m_{1}\cdot T}\cdot T^{m_{8}}\cdot \varepsilon^{m_{2}}\cdot e^{\frac{m_{4}}{\varepsilon }}\cdot {\left(1+\varepsilon \right)}^{m_{5}\cdot T}\cdot e^{m_{6}\cdot \varepsilon }\cdot {\dot{\varepsilon }}^{m_{3}}\cdot {\dot{\varepsilon }}^{m_{7}\cdot T},$$

where σ_p_—metal resistance to plastic deformation (MPa), T—temperature of the deformed material (K), ε—deformation, $\dot{\varepsilon }$—strain rate (s^−1^), A—material constant (MPa), and m1 ÷ m8—coefficients (dimensionless).

In this work, the deformation resistance, denoted as σ_p_, of the alloy under study was determined through uniaxial compression tests of cylindrical specimens with a diameter of 10 mm and a length of 12 mm. These tests were conducted using a Gleeble 3800 plastometer located in the laboratory of physical simulation of plastic processing at the Czestochowa University of Technology, Poland, Czestochowa. The Gleeble 3800 plastometer enables testing under thermomechanical conditions representative of actual industrial processes. The experiments were carried out in a vacuum chamber (vacuum level of at least 1 × 10 mm Hg) while maintaining a constant temperature of the deformed sample.

The rheological studies were planned to determine the coefficients (A, m1 ÷ m9) of the constitutive Equation (1) for the rolling conditions of 6082 aluminum alloy rods on a three-high skew rolling mill.

The plastometric tests were conducted under the following conditions:

- Temperature: 350 °C, 450 °C, 500 °C;
- Strain rate: 0.1 s^−1^, 1 s^−1^, 10 s^−1^;
- Actual deformation: max. 1.1.

The selected temperature range was intended to optimize the alloy’s machinability. Within the temperature interval of 0.5 to 0.7 times the alloy’s melting temperature (T_test_ = 0.5 ÷ 0.7∙T_melt_), the metal experiences dynamic polygonization and dynamic recrystallization processes, which enables the processing of the material with high shear deformations.

The numerical simulation was conducted with the following parameters: an initial material temperature of 350 °C; a roll rotation speed of 100 rpm; a roll surface trajectory inclination angle of 15°; a friction coefficient of 0.4; and a thermal conductivity in the contact zone between the workpiece and the rolls of 20,000 W/m^2^∙K. The initial rod diameter was reduced from 45 mm to 30 mm, with the process occurring in three successive stages: the first stage involved a change from 45 mm to 40 mm, the second stage from 40 mm to 35 mm, and the third stage from 35 mm to 30 mm.

**Figure 1:**
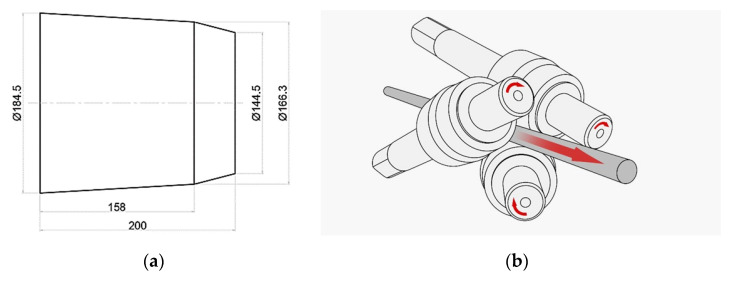
Geometrical parameters of the rolls (**a**) and their positioning within the working space of the rolling mill (**b**).

A triangular finite element mesh was used to spatially discretize the studied volume of material and tool. The geometric characteristics of the working rolls were also considered (Figure 1). The rolling mill parameters were set as follows: the tangential displacement of the roll axes relative to the rolling axis was 18 mm, the roll inclination angle to the rolling axis was 15°, and the roll drive power was 11 kW (for each roll). The analysis of the stress and deformation distributions within the rolled rod was performed for cross-sections I, II, and III (corresponding to the entrance, middle, and exit of the deformation zone).

The rolling process was conducted in three consecutive passes, reducing the workpiece diameter by 5 mm per pass. This enabled producing a final product with a diameter of 30 mm. The actual dimensions of the workpieces were measured after each pass using a caliper and a micrometer with an accuracy of ±0.01 mm. Following the rolling operation, samples were selected for microstructural analysis, hardness measurement, and comparative evaluation before and after the plastic deformation.

Hardness measurements were conducted at the Engineering Center (Shakarim University, Semey, Kazakhstan) using the HLV-1DT device (Shanghai Hualong Test Instruments Corporation, Shanghai, China), which operated according to the Vickers method. The measurements involved an indenter load of 1 N and a hold time of 10 s, in compliance with GOST 9450-76. The final hardness value was calculated as the average of ten measurements for each sample. The material exhibited stable hardness characteristics typical of 6082-series aluminum alloys in the initial state.

For the analysis, the samples were cut transversely using a G5012W band saw machine (“Tengzhou Uni-Tech Co”, Tengzhou, China) to a size of 15 mm. After cutting, the end surfaces of the samples were pre-ground and polished on a GP-1A single-disc grinding and polishing machine (“Trojan Material Technology Co.”, Suzhou, China). Sandpaper with a grain size ranging from P600 to P2500 was utilized for processing, enabling the achievement of an initial average surface roughness of 0.45 μm.

To reveal the microstructure, the surface was etched in a solution containing 15% concentrated sulfuric acid and 85% distilled water, following the method presented in the literature [29]. The etching was performed at a temperature between 60 and 70 °C for 30 s. Temperature control was maintained using a GM1500 infrared thermometer (“Benetech”, Shenzhen, China).

**Figure 2:**
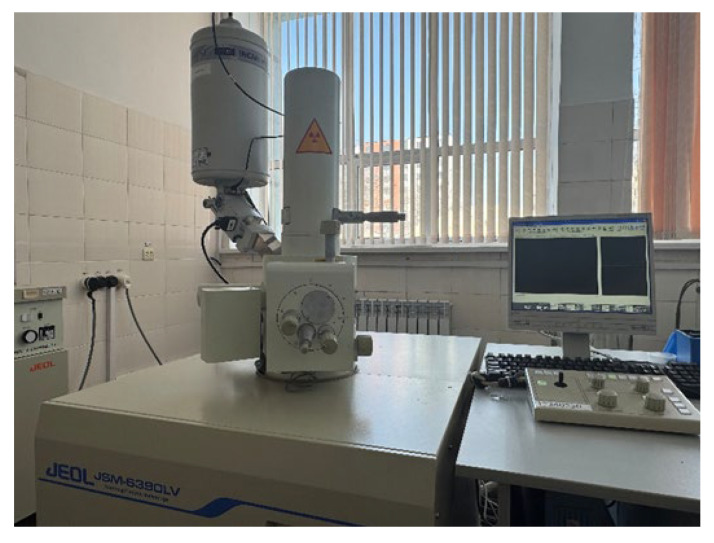
Scanning electron microscope JSM-6390 LV JEOL (Tokyo, Japan).

The microstructure of aluminum alloy 6082 before and after plastic deformation was analyzed at the Radioecological Research Laboratory of Shakarim University (Semey, Kazakhstan) using a low-vacuum scanning electron microscope JSM-6390 LV JEOL (Tokyo, Japan) (Figure 2).

## 3. Results and Discussion

**Figure 3:**
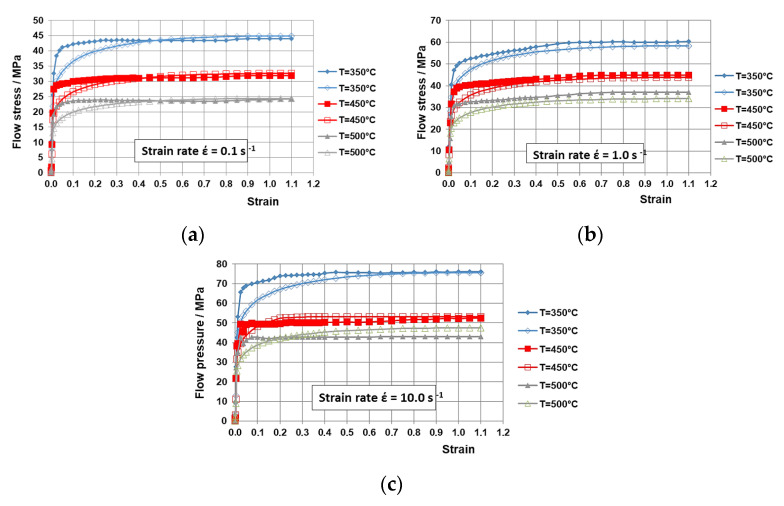
Flow curves σp − ε of Al 6082 alloy within the temperature range of 350 °C to 500 °C: (**a**) at a strain rate of 0.1 s^−1^, (**b**) at a strain rate of 1 s^−1^, (**c**) at a strain rate of 10 s^−1^; filled symbols are experimental curves, and empty symbols are approximated curves.

The deformation resistance flow curves (σ_p_ − ε) for the Al 6082 alloy, both actual and approximated, are presented in Figure 3. These curves depict the deformation resistance characteristics within a temperature range of 350 °C to 500 °C and a strain-rate range of 0.1 s^−1^ to 10 s^−1^.

The analysis of the flow curves σ_p_ − ε reveals that increasing the deformation temperature from 350 °C to 500 °C results in a reduction in the metal’s resistance to plastic deformation σ_p_. As shown in Figure 3, at low strain rates (Figure 3a) of 0.1 s^−1^, the material exhibits intensive strengthening, but it eventually reaches a “plateau” on the σ_p_ − ε flow curves within the studied deformation range. Conversely, at higher strain rates of 1 s^−1^ (Figure 3b) and 10 s^−1^ (Figure 3c), the rapid strain rate does not allow the recovery processes to counterbalance the strengthening processes, leading to a slight monotonic increase in some of the σ_p_ − ε flow curves.

As the temperature T increases, the influence of the strain rate becomes more pronounced due to an increase in the strain-rate sensitivity coefficient. This effect is particularly noticeable in the temperature range of 450 °C to 500 °C. Within this temperature range, an increase in the strain rate from 0.1 s^−1^ to 1.0 s^−1^ leads to a rise in the deformation resistance by approximately 35%, while an increase from 0.1 s^−1^ to 10 s^−1^ results in a 50% increase in the deformation resistance. In contrast, in the temperature range from 350 °C to 450 °C, an increase in the strain rate from 0.1 s^−1^ to 1.0 s^−1^ causes a 25% rise in the deformation resistance σ_p_. Furthermore, within the strain-rate range of 0.1 s^−1^ to 10 s^−1^, the increase in the deformation resistance σ_p_ was around 40%.

The approximation of the flow curves for the studied material has achieved an average error of no more than 10%. Assuming the function coefficients (A and m1–m8) are sufficiently well chosen such that the average approximation error does not exceed 15% [7], it can be concluded that the results obtained from this approximation process can be utilized in the numerical modeling of the SRM rolling operation under the specified thermomechanical processing conditions.

The coefficients of the function describing the metal’s resistance to plastic deformation were determined for the studied range of strain rates (0.1 s^−1^, 1 s^−1^, and 10 s^−1^) and temperatures from 350 °C to 500 °C. The obtained coefficient values of the function (1) are presented in Table 3.

**Figure 4:**
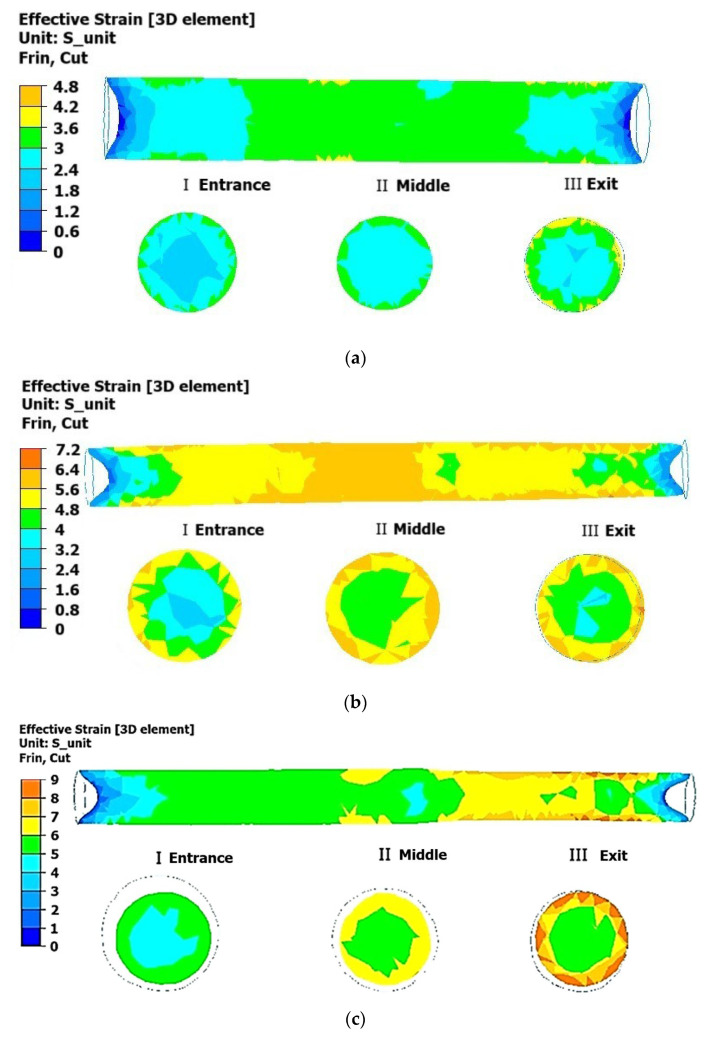
Distribution of accumulated plastic deformation in the longitudinal and transverse sections of a deformed rod at a temperature of 350 °C: (**a**) first pass (from 45 mm to 40 mm); (**b**) second pass (from 40 mm to 35 mm); (**c**) third pass (from 35 mm to 30 mm).

**Figure 5:**
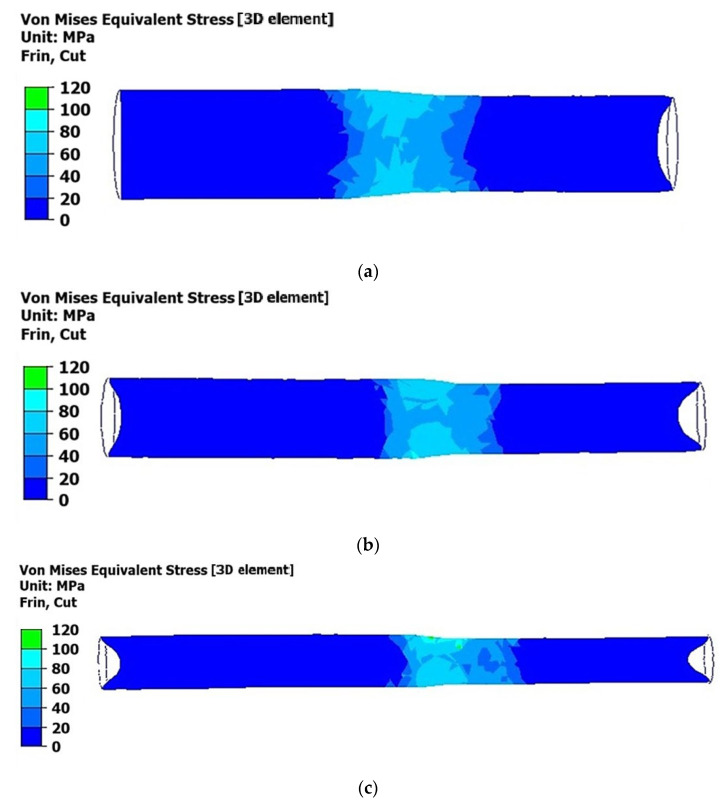
Distribution of equivalent stresses according to von Mises in the longitudinal section of a deformed rod at a temperature of 350 °C: (**a**) first pass (from 45 mm to 40 mm); (**b**) second pass (from 40 mm to 35 mm); (**c**) third pass (from 35 mm to 30 mm).

**Figure 6:**
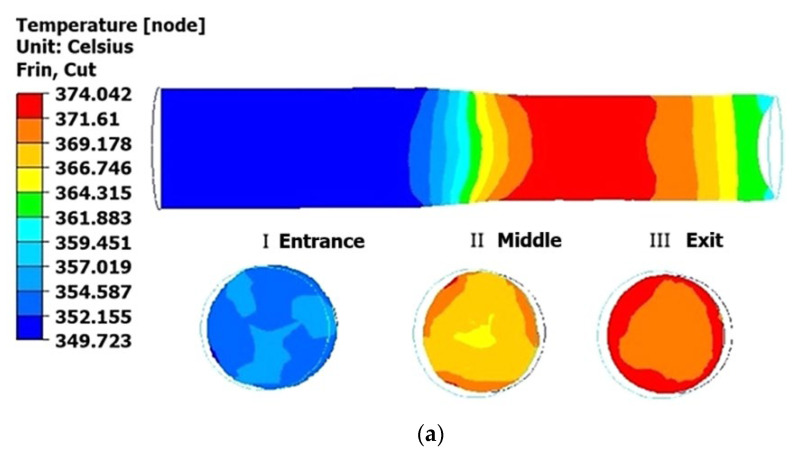

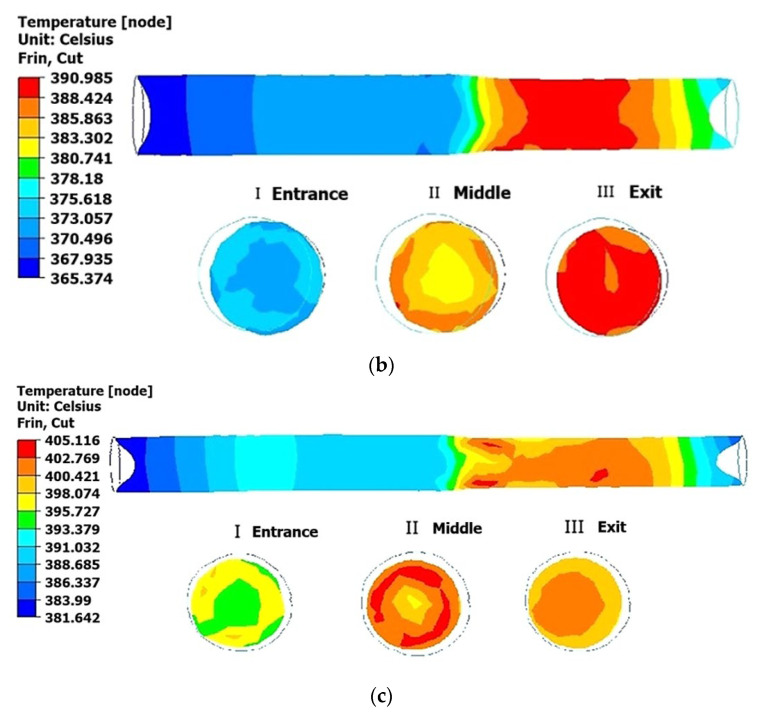
Temperature distribution across longitudinal and cross-sectional planes of the deformed rods at a temperature of 350 °C: (**a**) first pass (from 45 mm to 40 mm); (**b**) second pass (from 40 mm to 35 mm); (**c**) third pass (from 35 mm to 30 mm).

**Figure 7:**
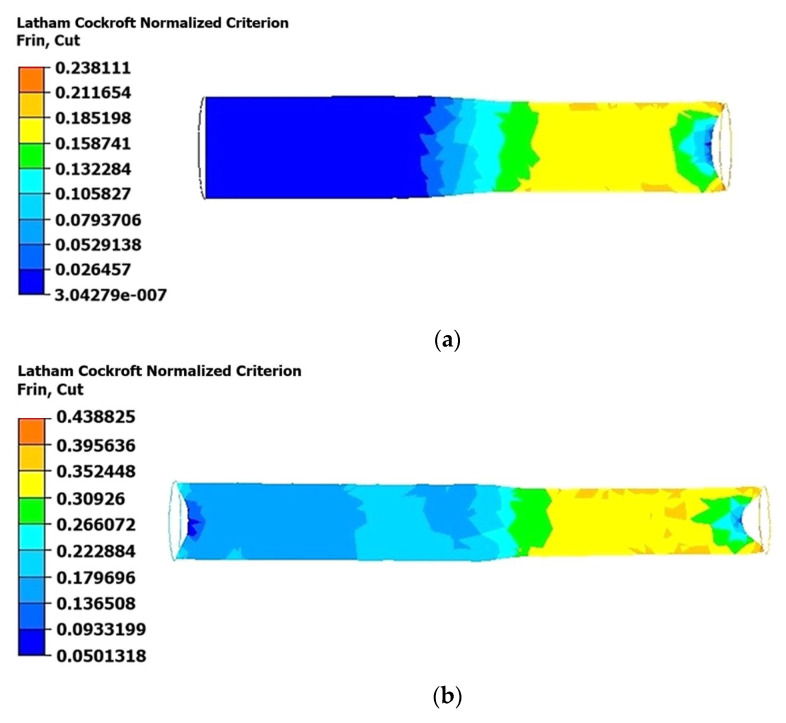

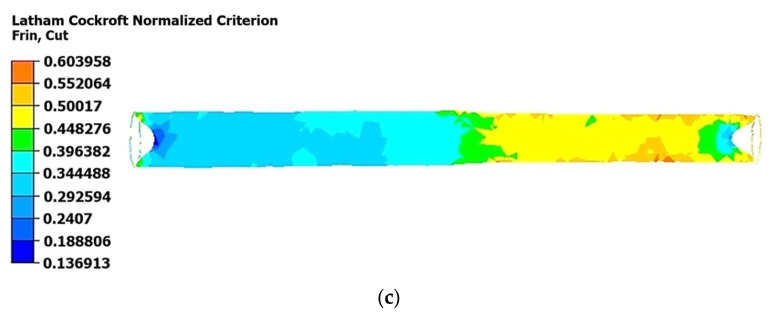
Distribution of the normalized Latham–Cockcroft criterion obtained as a result of numerical modeling in longitudinal sections of a deformed bar at a temperature of 350 °C: (**a**) first pass (from 45 mm to 40 mm); (**b**) second pass (from 40 mm to 35 mm); (**c**) third pass (from 35 mm to 30 mm).

The obtained data provide the basis for conducting numerical modeling of the rolling process on a three-high RSP mill using the specified initial conditions. Numerical modeling of alloy 6082 was performed using the ForgeNxT 2.1 software package [30], and the results are presented in Figure 4, Figure 5, Figure 6 and Figure 7.

ForgeNxT 2.1, a finite element-based commercial software, was utilized for simulating metal-forming processes. The analysis of metal flow during three-high skew rolling was performed using this software at the Faculty of Production Engineering and Materials Technology of the Czestochowa University of Technology (Poland).

The analysis of the data presented in Figure 4 reveals a gradual increase in the degree of deformation with each rolling pass. During the first pass (Figure 4a), the deformation was minimal, not exceeding five units, and concentrated primarily in the surface layers of the material. In the second pass (Figure 4b), the deformation increased to seven units, with its distribution becoming more uniform along the entire length of the rod. In the third pass (Figure 4c), the maximum deformation values were reached, up to nine units, and the distribution became even more uniform. Notably, the internal areas of the metal demonstrated a significantly lower degree of deformation, indicating a less intense impact on the central part of the material.

The near-surface layers of the rod (areas of the material adjacent to the surface) exhibited a higher maximum equivalent von Mises stress of approximately 80 MPa compared to the central part, where the equivalent stress averaged around 60 MPa, as shown in Figure 5a,b. This indicated a greater intensity of compressive stresses in the near-surface layers compared to the central part, where tensile stresses were present.

Specifically, Figure 5c reveals that the stress in the near-surface layers reached about 120 MPa, while in the central part of the rod, it ranged from 80 to 100 MPa. Generally, the near-surface layers of the rod demonstrate a higher degree of strain hardening compared to the central part, which can be attributed to the redistribution of plastic deformation during the process.

The analysis of the temperature fields revealed a gradual increase in the metal temperature throughout the rolling process. During the first pass (Figure 6a), the surface temperature of the bar reached approximately 374 °C, representing a 24 °C rise from the initial value. The second pass (Figure 6b) resulted in a further temperature increase to approximately 391 °C, 17 °C higher than the first pass. In the third pass (Figure 6c), the temperature reached approximately 405 °C, a 14 °C increment compared to the second pass. Consequently, the material temperature consistently increased with each subsequent rolling pass. The findings regarding the thermal effects of plastic deformation during the rolling process align with the data acquired through compression testing of samples using a Gleeble 3800 plastometer.

As shown in Figure 7, the normalized Latham–Cockcroft criterion values reached higher levels in the near-surface layers (zones adjacent to the surface of the material) of the longitudinal section compared to the central part of the rod. During the first rolling pass (Figure 7a), the Latham–Cockcroft criterion exhibited its highest values in the surface layers, reaching approximately 0.26, while its level was considerably lower in the central part, not exceeding 0.13. This suggests the presence of significant shear deformations and a higher strain rate in the peripheral zone.

In the second pass (Figure 7b), the Latham–Cockcroft criterion exhibited a rise in its value, and its distribution pattern became more pronounced. Specifically, within the near-surface layers, the criterion values escalated to 0.48, while in the central zone they remained within the range of 0.22–0.26. This suggests an enhanced degree of microstructural refinement on the surface of the rod.

During the third pass (Figure 7c), the process of damage accumulation attained its peak levels. Within the near-surface regions, the criterion values ranged from 0.55 to 0.60. Concurrently, in the central parts of the rod, the criterion values fell within the 0.44 to 0.50 range. Consequently, the dynamics of the normalized Latham–Cockcroft criterion corroborate the tendency for stress concentration in the near-surface layers, with the stresses intensifying as the degree of plastic deformation increases.

**Figure 8:**
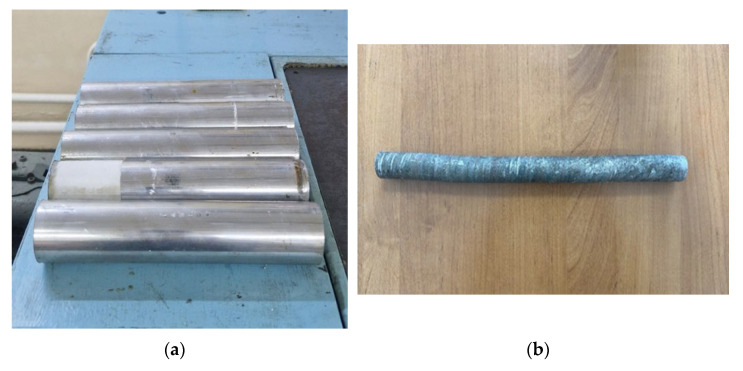
External appearance of blanks fabricated from aluminum alloy 6082: (**a**)—blank before rolling, (**b**)—blank after three-pass rolling.

An experiment on rolling alloy 6082 was conducted under laboratory conditions on a three-roll skew rolling mill, with parameters identical to those of the numerical simulation. The experiment was carried out at Kalsha LLC (Kazakhstan, Almaty). The appearance of the blanks is shown in Figure 8.

Before rolling, the blanks were preheated to 350 °C in a temperature-controlled muffle furnace. Temperature monitoring was performed using a thermocouple with an accuracy of ±5 °C. The roll rotation speed was kept constant throughout the deformation process.

During the deformation treatment, the geometric characteristics and surface quality of the workpieces were examined after each rolling pass. Analysis of the geometric dimensions revealed that the actual rod diameter was accurate to within ±0.25 mm. After the rolling process, a slight curvature was observed, which was then corrected using a bending press. The rod surface exhibited a trace characteristic of the screw movement of the metal from contact with the rollers. No cavities, folds, or other discontinuity defects were observed. A comparative analysis of the geometric parameters of rolled samples, obtained through empirical means and computational modeling, confirms that the employed simulation methodology was accurate and reliable.

**Figure 9:**
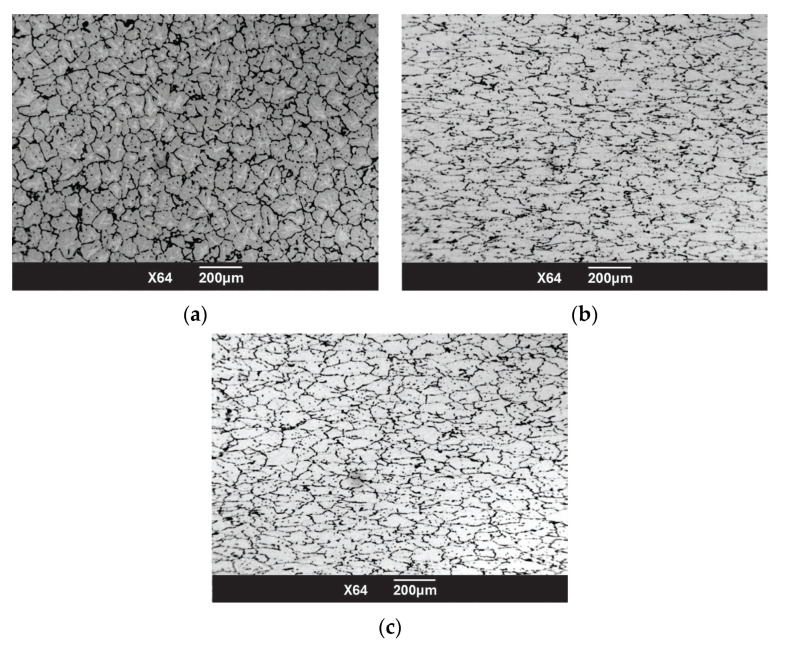
(**a**) Initial structure of alloy 6082; (**b**) structure after SRM processing in the central part; (**c**) structure on the periphery of the rod.

Microstructural studies revealed that the initial material had a uniform recrystallized structure with a grain size of no more than 80 µm (Figure 9a). After rolling, grain refinement was observed.

The analysis of Figure 9b,c confirms the findings of the numerical modeling, indicating more intense processing of the near-surface regions and the presence of tensile stresses in the central portion of the rod. In the central area, the grains exhibit a more elongated morphology (Figure 9b). Conversely, at the periphery, the grains are less elongated due to the combined effects of high deformations and elevated temperatures, accounting for the thermal influence (T ≈ 400 °C). In certain locations, single equiaxed grains with a maximum size of 10 μm are observed.

As a result of SPD process, a single heating and three rolling passes enabled significant refinement of the alloy’s microstructure, achieving the desired geometry without compromising continuity.

**Figure 10:**
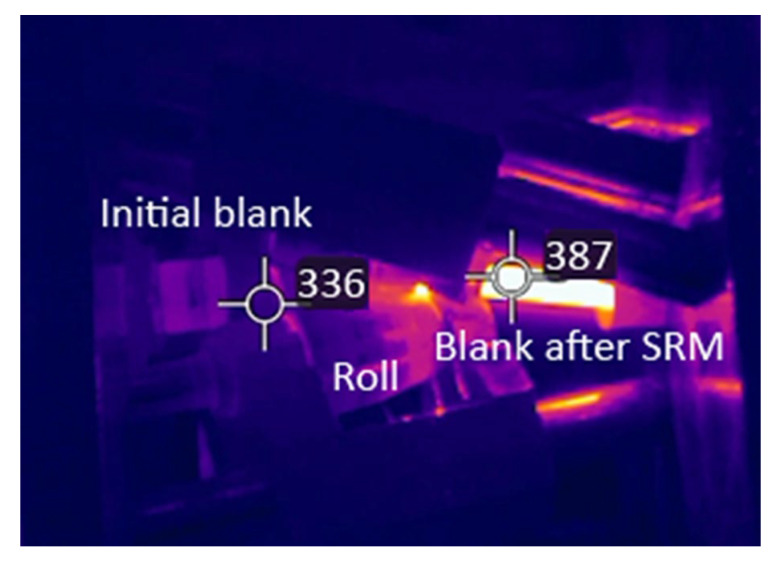
Results of temperature monitoring using a Flir T540 thermovisor during the first pass of rolling an alloy 6082 rod.

During the rolling process, the temperature was monitored in real time using a Flir T540 thermovisor (“Teledyne FLIR”, Wilsonville, OR, USA), which recorded temperature changes on the rod surface (Figure 10). The analysis of the results showed acceptable convergence with the computer model calculations. The initial temperature differences can be attributed to the time required to transfer the workpiece from the furnace to the rolling mill line. A more pronounced thermal effect was observed after rolling, with the temperature difference between the experimental and calculated values being no more than 10%.

Hardness measurements were conducted in the initial state and after rolling at 350 °C. The measurement results are presented in Table 4.

After rolling at 350 °C, an increase in hardness was observed, which can be attributed to grain refinement and an elevated density of crystal lattice defects. However, the hardness values exhibited slight variations. This can be explained by the occurrence of dynamic recrystallization within the metal structure during the rolling process, which led to the formation of single equiaxed grains in the surface layers of the rolled metal. The measurements revealed an uneven hardness distribution, correlating with the degree of accumulated deformation. Specifically, the surface layers exhibited slightly higher hardness compared to the central region of the rod.

In general, it can be noted that the selected rolling temperature regime allowed for continued rolling with large stretches in appropriate cases, without the need for additional heating. This is due to the technological reserve, as indicated by the analysis of the structure and hardness.

## 4. Conclusions

Based on the results of the work carried out, the following conclusions can be drawn:

1. Rheological studies were conducted on alloy 6082 using the uniaxial compression method on a Gleeble 3800 plastometer. For the first time, the influence of the deformation-velocity parameters of the SRM processing on the resistance to deformation of alloy 6082 in the temperature range of 350 ÷ 500 °C was established;
2. A numerical model of rolling was developed and verified with the results of a physical experiment in laboratory conditions. The verification results indicated acceptable convergence between the calculation results and the structure and properties of the rolled metal;
3. Numerical and real experiments were performed on rolling rods made of aluminum alloy 6082 on a three-high SRM, followed by analysis of the structure and properties of the rolled material;
4. From the perspective of the technological effectiveness of the alloy and the costs of heating the metal, it was noted that SPD at a heating temperature of T = 350 °C is advisable. Considering the intensive processing of the metal and the thermal effect of plastic deformation, dynamic softening processes occur in the metal, allowing up to three rolling passes to be carried out without additional heating;
5. The selected rolling modes enable significant refinement of the metal structure, ensuring the required geometry of the rod without compromising continuity;
6. The material and time efficiency of using modern means of substantiating the TMO of alloy 6082 through physical and numerical modeling methods was noted.

## Figures and Tables

**Table 1:** Chemical composition of the studied aluminum alloy (%).

Alloy	Si	Fe	Cu	Mn	Mg	Cr	Zn	Ti	Al
6082	0.95	≤0.18	≤0.02	0.50	0.95	≤0.03	≤0.02	≤0.02	R

**Table 2:** Initial parameters of the workpiece.

Parameter	Value
Alloy (aluminum)	6082
Diameter, mm	45
Length, mm	200
Rolling temperature, °C	350

**Table 3:** Parameters A and m_1_–m_8_ used to determine the σ_p_ value of Al 6082 alloy.

A	m1	m2	m3	m4	m5	m6	m7	m8
1.041582 × 10^−9^	−0.01578	0.3425125	−0.0116075	−0.0002532	−0.000226	−0.12197	0.000312	5.295665

**Table 4:** Results of hardness measurements of 6082 alloy rods in the initial state and after three-high SRM processing.

Measurement No.	Initial State (HV)	After Plastic Deformation at 350 °C (HV)
1	92.7 ± 2.7	98.1 ± 2.9
2	90.1 ± 2.7	96.4 ± 1.9
3	89.7 ± 2.6	95.8 ± 2.8
4	84.4 ± 2.5	87.4 ± 1.7
5	81.8 ± 2.4	85.8 ± 2.5
6	81.7 ± 2.4	87.6 ± 2.6
7	84.5 ± 1.6	86.8 ± 1.7
8	89.5 ± 1.7	88.8 ± 2.6
9	90.0 ± 2.6	93.9 ± 2.8
10	92.8 ± 2.7	94.8 ± 2.8
Average value	87.7	91.5

## Data Availability

The original contributions presented in this study are included in the article. Further inquiries can be directed to the corresponding author.

## References

[1] Tomstad A.J., Thomesen S., Børvik T., Hopperstad O.S. (2021). Effects of constituent particle content on ductile fracture in isotropic and anisotropic 6000-series aluminium alloys. Mater. Sci. Eng. A.

[2] Bhat K.U., Panemangalore D.B., Kuruveri S.B., John M., Menezes P.L. (2022). Surface modification of 6xxx Series aluminum alloys. Coatings.

[3] Poznak A., Freiberg D., Sanders P. (2018). Automotive wrought aluminium alloys. Fundamentals of Aluminium Metallurgy.

[4] Poletti C., Bureau R., Loidolt P., Simon P., Mitsche S., Spuller M. (2018). Microstructure evolution in a 6082 aluminium alloy during thermomechanical treatment. Materials.

[5] Segal V. (2018). Review: Modes and Processes of Severe Plastic Deformation (SPD). Materials.

[6] Qi Y., Kosinova A., Lakin E., Popov V.V., Rabkin E., Lapovok R. (2019). Effect of SPD Processing on the Strength and Conductivity of AA6061 Alloy. Adv. Eng. Mater..

[7] Awasthi A., Gupta A., Saxena K.K., Diwedi R.K. (2022). Equal channel angular processing on aluminium and its alloys—A review. Mater. Today Proc..

[8] Berndt N., Reiser N.A., Wagner M.F.X. (2025). Evolution of plastic deformation during multi-pass ECAP of an AA6060 aluminum alloy–An experimental flow line analysis. J. Mater. Res. Technol..

[9] Agarwal K.M., Tyagi R.K., Choubey V., Saxena K.K. (2022). Mechanical behaviour of Aluminium Alloy AA6063 processed through ECAP with optimum die design parameters. Adv. Mater. Process. Technol..

[10] Safari M., Joudaki J. (2020). Effect of temperature on strength and hardness in multi-pass equal channel angular pressing (ECAP) of aluminum alloys. Trans. Indian Inst. Met..

[11] Segal V.M. (1995). Materials processing by simple shear. Mater. Sci. Eng. A.

[12] Valiev R. (2004). Nanostructuring of metals by severe plastic deformation for advanced properties. Nat. Mater..

[13] Gazizov M., Telesov V., Zakharov V.V., Kaibyshev R. (2011). Effect of ECAP on mechanical properties of an Al-Cu-Mg-Ag-Sc alloy. Materials Science Forum.

[14] Shaban M., Alsunaydih F.N., Kouta H., El-Sanabary S., Alrumayh A., Alateyah A.I., Alawad M.O., El-Garaihy W.H., El-Taybany Y. (2024). Optimization of wear parameters for ECAP-processed ZK30 alloy using response surface and machine learning approaches: A comparative study. Sci. Rep..

[15] Mazilkin A.A., Kogtenkova O.A., Straumal B.B., Valiev R., Baretzky B. (2005). Formation of nanostructure during high-pressure torsion of Al-Zn, Al-Mg and Al-Zn-Mg alloys. Defect and Diffusion Forum.

[16] Straumal B.B., Mazilkin A.A., Protasova S.G., Goll D., Baretzky B., Bakai A.S., Dobatkin S.V. (2011). Formation of two amorphous phases in the Ni60Nb18Y22 alloy after high pressure torsion. Kovove Mater. Metall. Mater.

[17] Wang F., Ding C., Yang Z., Zhang H., Ding Z., Li H., Xu J., Shan D., Guo B. (2024). Microstructure Evolution and Mechanical Properties of AlCoCrFeNi2. 1 Eutectic High-Entropy Alloys Processed by High-Pressure Torsion. Materials.

[18] Xie K., Wang H., Yin D., Atrens A., Zhao M.C. (2024). Evolution of microstructure and texture and their influence on the strength of an accumulative roll bonded (ARBed) Al-Sc-Zr-Er-Ti alloy. J. Mater. Res. Technol..

[19] Długosz P., Bochniak W., Ostachowski P., Molak R., Duarte Guigou M., Hebda M. (2021). The influence of conventional or KOBO extrusion process on the properties of AZ91 (MgAl9Zn1) alloy. Materials.

[20] Salishchev G.A., Zherebtsov S.V., Galeyev R.M. (2002). Evolution of Microstructure and Mechanical Behavior of Titanium During Warm Multiple Deformation. Ultrafine Grained Materials II.

[21] Beygelzimer Y., Varyukhin V., Orlov D., Efros B., Stolyarov V., Salimgareyev H. Microstructural evolution of titanium under twist extrusion. Proceedings of the TMS Annual Meeting.

[22] Schindler I., Kawulok P., Očenášek V., Opěla P., Kawulok R., Rusz S. (2019). Flow stress and hot deformation activation energy of 6082 aluminium alloy influenced by initial structural state. Metals.

[23] Kawałek A., Dyja H., Gałkin A.M., Ozhmegov K.V., Sawicki S. (2014). Physical modelling of the plastic working processes of zirconium alloy bars and tubes in thermomechanical conditions. Arch. Metall. Mater..

[24] Dyja H., Sobczak K., Kawałek A., Knapiński M. (2013). The analysis of the influence of varying types of shape grooves on the behavior of internal material discontinuities during rolling. Metalurgija.

[25] Kawałek A., Dyja H., Galkin A.M., Ozhmegov K.V., Knapiński M. (2015). Physical modelling of the plastic working processes of modified Zr-Nb zirconium alloy bars and tubes. Metalurgija.

[26] Kawałek A., Gałkin A., Dyja H., Ozhmegov K., Knapiński M., Koczurkiewicz B. (2015). Plastometric modelling of the E635M zirconium alloy multistage forging process. Solid State Phenom..

[27] Dyja H., Kawalek A., Sawicki S., Laber K., Ozhmegov K. Physical modelling of the zirconium alloy tube pilger rolling process. Proceedings of the METAL 2017—26th International Conference on Metallurgy and Materials.

[28] Henzel A., Shpittel T. (1982). Raschetjen Ergosilovyh Parametrov V Processah Obrabotki Metallov Davleniem: Spravochnik [Calculation of Energy—Power Parameters in Metal Forming Processes. Directory].

[29] Gerasimova L.P., Guk Y.P. (2017). Prakticheskaya Metallografiya [Practical Metallography].

[30] (2008). Forge 2008 3D Forging Simulation Software: Rheology and Interfaces Module, FPDBase Version 1.3.

